# Security Analysis of the Unrestricted Identity-Based Aggregate Signature Scheme

**DOI:** 10.1371/journal.pone.0128081

**Published:** 2015-05-18

**Authors:** Kwangsu Lee, Dong Hoon Lee

**Affiliations:** Center for Information Security Technologies, Korea University, Seoul, Korea; Nankai University, CHINA

## Abstract

Aggregate signatures allow anyone to combine different signatures signed by different signers on different messages into a short signature. An ideal aggregate signature scheme is an identity-based aggregate signature (IBAS) scheme that supports full aggregation since it can reduce the total transmitted data by using an identity string as a public key and anyone can freely aggregate different signatures. Constructing a secure IBAS scheme that supports full aggregation in bilinear maps is an important open problem. Recently, Yuan et al. proposed such a scheme and claimed its security in the random oracle model under the computational Diffie-Hellman assumption. In this paper, we show that there is an efficient forgery on their IBAS scheme and that their security proof has a serious flaw.

## 1 Introduction

Aggregate signature schemes allow anyone to combine *n* different signatures on different *n* messages signed by different *n* signers into a short aggregate signature. The main advantage of aggregate signature schemes is the reduction of signature communication and storage requirements by the compression of multiple signatures into a single one. The applications of aggregate signature schemes include secure routing protocols, public-key infrastructure systems, and sensor networks. Boneh et al. [1] proposed the first aggregate signature scheme allowing anyone to combine different signatures in bilinear groups and proved its security in the random oracle model. Subsequently Lysyanskaya et al. [2] constructed a sequential aggregate signature scheme in which signatures can be combined sequentially, and Gentry and Ramzan [3] proposed a synchronized aggregate signature scheme in which all signers should share synchronized information. There are many additional aggregate signature schemes with different properties [4–13].

Although aggregate signature schemes can reduce the size of signatures by their aggregating them, they usually cannot reduce the total amount of transmitted data significantly since a verifier should additionally retrieve the public keys of all signers. Therefore, an important issue in aggregate signature schemes is reducing the size of public keys [7, 10–12]. An ideal solution to this problem is the use of an identity-based aggregate signature (IBAS) scheme since it uses an already known identity string as a user’s public key [3]. However, there is only one IBAS scheme with full aggregation proposed by Hohenberger et al. [9] in multilinear maps. Multilinear maps are attractive tools for cryptographic constructions, but they are currently impractical since they are based on leveled homomorphic encryption schemes [14]. There are some IBAS schemes in bilinear maps, but those schemes only support sequential or synchronized aggregation [3, 6, 8]. Therefore, constructing an IBAS scheme featuring full aggregation in bilinear maps is an important open problem.

The main challenge when devising an IBAS scheme with full aggregation is aggregating the random values of all signers such that each one hides the private key in the signature [3]. For this reason, current IBAS schemes can only aggregate the randomness of all signers through synchronized or sequential aggregation [3, 6]. Additionally, designing a secure IBAS scheme is not an easy task. In fact, even the original IBAS scheme of Boldyreva et al. [6] was broken by Hwang et al. [15] and had to be corrected later. Recently, Yuan et al. [16] proposed an IBAS scheme featuring full aggregation in bilinear maps and claimed its security in random oracle models. The authors first proposed an IBS scheme in bilinear maps and from that constructed an IBAS scheme. To demonstrate the security of their IBS scheme, the authors claimed that its security could be proven under the computational Diffie-Hellman (CDH) assumption by using Forking Lemma in the random oracle model.

In this paper, we show that the IBS and IBAS schemes of Yuan et al. [16] are not secure at all. First, we show that there is a universal forgery algorithm against their IBS scheme which outputs a forged signature by using two valid signatures. This forgery algorithm also applies to their IBAS scheme. One may wonder whether our forgery algorithm contradicts the security claims of their schemes or not. To explain this, we then show that the security proof of Yuan et al.’s IBS scheme has a serious flaw. The proof essentially relies on the condition that two forged signatures obtained using Forking Lemma have the same randomness. However, we reveal that the forged signatures of an adversary cannot satisfy this condition since the signatures of Yuan et al.’s IBS scheme are publicly re-randomized.

This paper is organized as follows: In Section 2, we review bilinear groups and the IBS and IBAS schemes of Yuan et al. In Section 3, we present a universal forgery against the IBS scheme. In Section 4, we analyze the security proof of Yuan et al.’s IBS scheme and show that it has a serious flaw.

## 2 The IBAS Scheme of Yuan et al

In this section, we review bilinear groups, complexity assumptions, and the IBS and IBAS schemes of Yuan et al. [16]. For more details, refer to [16].

### 2.1 Bilinear Groups and Complexity Assumptions

Let 𝔾 and 𝔾_*T*_ be two multiplicative cyclic groups of prime order *p*. Let *g* be a generator of 𝔾. A bilinear map is a map *e*:𝔾 × 𝔾 → 𝔾_*T*_ with the following properties:

1. Bilinearity: for all *u*, *v* ∈ 𝔾 and all *a*, *b* ∈ ℤ_*p*_, we have *e*(*u* ^*a*^, *v* ^*b*^) = *e*(*u*, *v*)^*ab*^.
2. Non-degeneracy: *e*(*g*, *g*) ≠ 1.

We say that 𝔾 is a bilinear group if the group operations in 𝔾 and 𝔾_*T*_ as well as the bilinear map *e* are all efficiently computable.

**Assumption 2.1** (Computational Diffie-Hellman, CDH). *Let 𝔾 be a cyclic group of prime order p and g be a generator of 𝔾. The CDH assumption is that if the challenge tuple $D=(p,𝔾,g,g^{a},g^{b})$ is given, no probabilistic polynomial-time (PPT) algorithm 𝓐 can compute g^ab^ ∈ 𝔾 with more than a negligible advantage. The advantage of 𝓐 is defined as ${\text{Adv}}_{𝓐}^{CDH}(\lambda )=\text{Pr}[𝓐(D)=g^{ab}]$ where the probability is taken over random choices of a,b ∈ ℤ_p_.*

### 2.2 The Identity-Based Signature Scheme

The IBS scheme of Yuan et al. [16] consists of the algorithms, **Setup**, **GenKey**, **Sign**, and **Verify**, which are described as follows:

- **Setup**(1^*λ*^): This algorithm takes as input a security parameter 1^*λ*^. It generates bilinear groups 𝔾, 𝔾_*T*_ of prime order *p*. Let *g* be a random generator of 𝔾. It chooses random exponents $s_{1},s_{2}\in {\mathbb{Z}}_{p}^{*}$ and two cryptographic hash functions *H*_1_:{0,1}* → 𝔾 and $H_{2}:{\left\{0,1\right\}}^{*}\to {\mathbb{Z}}_{p}^{*}$. It outputs a master key *MK* = (*s*_1_, *s*_2_) and public parameters $PP=((p,𝔾,{𝔾}_{T},e),\;g,\;g_{1}=g^{s_{1}},g_{2}=g^{s_{2}},\;H_{1},H_{2})$.
- **GenKey**(*ID*, *MK*, *PP*): This algorithm takes as input an identity *ID* ∈ {0,1}*, the master key *MK* = (*s*_1_, *s*_2_), and the public parameters *PP*. It outputs a private key $SK_{ID}=(D_{1}=H_{1}{\left(ID\right)}^{s_{1}},\;D_{2}=H_{1}{\left(ID\right)}^{s_{2}})$.
- **Sign**(*M*, *SK*_*ID*_, *PP*): This algorithm takes as input a message *M* ∈ {0,1}*, a private key *SK*_*ID*_ = (*D*_1_, *D*_2_), and the public parameters *PP*. It selects a random exponent $r\in {\mathbb{Z}}_{p}^{*}$ and computes *h* = *H*_2_(*ID*‖*M*). It outputs a signature $\sigma =(U=g^{r},\;V=D_{1}^{h}\cdot g_{1}^{r},\;W=D_{2}\cdot g_{2}^{r})$.
- **Verify**(*σ*, *ID*, *M*, *PP*): This algorithm takes as input a signature *σ* = (*U*, *V*, *W*), an identity *ID* ∈ {0,1}*, a message *M*{0,1}*, and the public parameters *PP*. It computes *h* = *H*(*ID*‖*M*) and checks whether $e(V,g)\overset{?}{=}e(H_{1}{\left(ID\right)}^{h}\cdot U,g_{1})$ and $e(W,g)\overset{?}{=}e(H_{1}(ID)\cdot U,g_{2})$. If both equations hold, then it outputs 1. Otherwise, it outputs 0.

**Claim 2.2** ([16]) *The above IBS scheme is existentially unforgeable under chosen message attacks in the random oracle model if the CDH assumption holds.*

**Remark 2.3** *The original IBS and IBAS schemes of Yuan et al. are described in the additive notation in bilinear groups. However, in this paper, we use the multiplicative notation for notational simplicity.*

**Remark 2.4** *The signatures of Yuan et al.’s IBS scheme are publicly re-randomized. If σ = (U, V, W) is a valid signature, then a re-randomized signature* $\sigma^{\prime }=(U\cdot g^{r^{\prime }},V\cdot g_{1}^{r^{\prime }},W\cdot g_{2}^{r^{\prime }})$ *is also valid where r′ is a random exponent in* ${\mathbb{Z}}_{p}^{*}$.

### 2.3 The Identity-Based Aggregate Signature Scheme

The IBAS scheme consists of the algorithms, **Setup**, **GenKey**, **Sign**, **Verify**, **Aggregate**, and **AggVerify**. The **Setup**, **GenKey**, **Sign**, and **Verify** algorithms of Yuan et al.’s IBAS scheme are the same as their IBS scheme counterparts. The additional algorithms of the IBAS scheme of Yuan et al. [16] are described as follows:

- **Aggregate**(*σ*_1_, *σ*_2_, *S*_1_, *S*_2_, *PP*): This algorithm takes as input a signature *σ*_1_ = (*U*_1_, *V*_1_, *W*_1_) on a multiset *S*_1_ = {(*ID*_1,1_, *M*_1,1_), …, (*ID*_1, *n*_1__, *M*_1, *n*_1__)} of identity and message pairs, a signature *σ*_2_ = (*U*_2_, *V*_2_, *W*_2_) on a multiset *S*_2_ = {(*ID*_2,1_, *M*_2,1_), …, (*ID*_2, *n*_2__, *M*_2, *n*_2__)} of identity and message pairs, and the public parameters *PP*. It outputs an aggregate signature $\sigma =(U=U_{1}\cdot U_{2},V=V_{1}\cdot V_{2},W=W_{1}\cdot W_{2})$ on the multiset *S* = *S*_1_∪*S*_2_.
- **AggVerify**(*σ*, *S*, *PP*): This algorithm takes as input an aggregate signature *σ* = (*U*, *V*, *W*), a multiset *S* = {(*ID*_1_, *M*_1_), …, (*ID*_*n*_, *M*_*n*_)} of identity and message pairs, and the public parameters *PP*. It computes *h*_*i*_ = *H*(*ID*_*i*_‖*M*_*i*_) for *i* = 1, …, *n* and checks whether $e(V,g)\overset{?}{=}e(\prod_{i=1}^{n}H_{1}{\left(ID_{i}\right)}^{h_{i}}\cdot U,g_{1})$ and $e(W,g)\overset{?}{=}e(\prod_{i=1}^{n}H_{1}(ID_{i})\cdot U,g_{2})$. If both equations hold, then it outputs 1. Otherwise, it outputs 0.

**Claim 2.5** ([16]) *The above IBAS scheme is existentially unforgeable under chosen message attacks in the random oracle model if the underlying IBS scheme is unforgeable under chosen message attacks.*

## 3 Forgery Attacks on the IBAS Scheme

In this section, we show that the IBS and IBAS schemes of Yuan et al. are not secure at all by presenting an efficient forgery algorithm. In fact, our forgery algorithm is universal since anyone who has two valid signatures on the same identity and different messages can generate a forged signature on the same identity and any message of its choice.

**Lemma 3.1** *There is a probabilistic polynomial-time (PPT) algorithm ℱ that can forge the IBS scheme of Yuan et al. with non-negligible probability if ℱ makes just two signature queries.*

*Proof*. The basic idea of our forgery attack is that if a forger obtains two valid signatures on an identity, then he can derive another valid signature by computing a linear combination of those signatures with carefully chosen scalar values. A forgery algorithm ℱ is described as follows:

1. ℱ randomly selects a target identity ID* and two different messages *M*_1_ and *M*_2_. It obtains a signature *σ*_1_ = (*U*_1_, *V*_1_, *W*_1_) on the pair (ID*, *M*_1_) and a signature *σ*_2_ = (*U*_2_, *V*_2_, *W*_2_) on the pair (ID*, *M*_2_) from the signing oracle.
2. It randomly selects a target message M* for a forged signature. Next, it computes *h*_1_ = *H*_2_(ID*‖*M*_1_), *h*_2_ = *H*_2_(ID*‖*M*_2_), and h* = *H*_2_(ID*‖M*). It computes two exponents *δ*_1_, *δ*_2_ that satisfy the following equation
  $$\begin{array}{c} {}[\begin{array}{cc} h_{1} & h_{2}\\ 1 & 1 \end{array}][\begin{array}{c} \delta_{1}\\ \delta_{2} \end{array}]=[\begin{array}{c} h^{*}\\ 1 \end{array}]\;\mathrm{mod}\;p. \end{array}$$
  Note that *δ*_1_ and *δ*_2_ can be computed using linear algebra since the determinant *h*_1_−*h*_2_ of the left matrix is not zero if *h*_1_ ≠ *h*_2_.
3. Finally, ℱ outputs a forged signature σ* on the identity and message pair (ID*, M*) as
  $$\begin{array}{c} \sigma^{*}=(U^{*}=U_{1}^{\delta_{1}}\cdot U_{2}^{\delta_{2}},\;V^{*}=V_{1}^{\delta_{1}}\cdot V_{2}^{\delta_{2}},\;W^{*}=W_{1}^{\delta_{1}}\cdot W_{2}^{\delta_{2}}). \end{array}$$

To finish the proof, we should show that the forger ℱ outputs a (forged) signature with non-negligible probability and that the forged signature passes the verification algorithm. We know that ℱ always outputs a signature if *h*_1_ ≠ *h*_2_. Because *H*_2_ is a collision-resistant hash function and *M*_1_ ≠ *M*_2_, *h*_1_ ≠ *h*_2_ is of non-negligible probability. Now we should show that the forged signature is correct according to the verification algorithm. Let *r*_1_ and *r*_2_ be the randomness of *σ*_1_ and *σ*_2_ respectively. The correctness of the forged signature is easily verified as follows:

$$\begin{array}{ccc} U^{*} & = & U_{1}^{\delta_{1}}\cdot U_{2}^{\delta_{2}}=g^{r_{1}\delta_{1}+r_{2}\delta_{2}}=g^{r^{*}},\\ V^{*} & = & V_{1}^{\delta_{1}}\cdot V_{2}^{\delta_{2}}={\left(H_{1}{\left(ID^{*}\right)}^{s_{1}h_{1}}g_{1}^{r_{1}}\right)}^{\delta_{1}}\cdot {\left(H_{1}{\left(ID^{*}\right)}^{s_{1}h_{2}}g_{1}^{r_{2}}\right)}^{\delta_{2}}\\ & = & H_{1}{\left(ID^{*}\right)}^{s_{1}\left(h_{1}\delta_{1}+h_{2}\delta_{2}\right)}g_{1}^{r_{1}\delta_{1}+r_{2}\delta_{2}}=H_{1}{\left(ID^{*}\right)}^{s_{1}h^{*}}g_{1}^{r^{*}},\\ W^{*} & = & W_{1}^{\delta_{1}}\cdot W_{2}^{\delta_{2}}={\left(H_{1}{\left(ID^{*}\right)}^{s_{2}}g_{2}^{r_{1}}\right)}^{\delta_{1}}\cdot {\left(H_{1}{\left(ID^{*}\right)}^{s_{2}}g_{2}^{r_{2}}\right)}^{\delta_{2}}\\ & = & H_{1}{\left(ID^{*}\right)}^{s_{2}\left(\delta_{1}+\delta_{2}\right)}g_{2}^{r_{1}\delta_{1}+r_{2}\delta_{2}}=H_{1}{\left(ID^{*}\right)}^{s_{2}}g_{2}^{r^{*}} \end{array}$$

where the randomness of the forged signature is defined as r* = *r*_1_ *δ*_1_+*r*_2_ *δ*_2_ *mod* *p*. This completes the proof.

**Corollary 3.2** *There is a PPT algorithm ℱ that can forge the IBAS scheme of Yuan et al. with non-negligible probability if ℱ makes just two signature queries.*

The proof of this corollary is trivial from the proof of the previous Lemma since the IBAS scheme uses the IBS scheme as its underlying signature scheme. We omit the proof.

## 4 Our Analysis of the Security Proof

From the forgery algorithm presented in the previous section, it is evident that the IBS and IBAS schemes of Yuan et al. are not secure. However, Yuan et al. [16] claimed that their IBS scheme is secure in the random oracle model under the CDH assumption. In this section, we analyze the security proof of Yuan et al. and show that it has a critical flaw.

### 4.1 The Original Proof

In this subsection, we briefly review the security proof of Yuan et al.’s IBS scheme [16] that solves the CDH problem by using Forking Lemma [17, 18].

Suppose there is an adversary 𝓐 that outputs a forged signature of the IBS scheme with a non-negligible advantage. A simulator ℬ that solves the CDH problem using 𝓐 when it is given a challenge tuple *D* = ((*p*, 𝔾, 𝔾_*T*_, *e*), *g*, *g* ^*a*^, *g* ^*b*^) is described as follows:

- **Setup**: ℬ chooses a random exponent $s_{2}\in {\mathbb{Z}}_{p}^{*}$ and maintains an *H*_1_-list and an *H*_2_-list for random oracles. It implicitly sets *s*_1_ = *a* and publishes the public parameters $PP=((p,𝔾,{𝔾}_{T},e),g,\;g_{1}=g^{a},g_{2}=g^{s_{2}},\;H_{1},H_{2})$.
- **Hash Query**: If this is an *H*_1_ hash query on an identity *ID*_*i*_, then ℬ handles this query as follows: If the identity *ID*_*i*_ already appears in the *H*_1_-list, then it responds with the value in the list. Otherwise, it picks a random coin *c* ∈ {0,1} with Pr[*c* = 0] = *δ* for some *δ* and proceeds as follows: If *c* = 0, then it chooses $t_{i}\in {\mathbb{Z}}_{p}^{*}$ and sets $Q_{i}={\left(g^{b}\right)}^{t_{i}}$. If *c* = 1, then it chooses $t_{i}\in {\mathbb{Z}}_{p}^{*}$ and sets $Q_{i}=g^{t_{i}}$. Next, it adds (*ID*_*i*_, *t*_*i*_, *c*, *Q*_*i*_) to the *H*_1_-list and responds to 𝓐 with *H*_1_(*ID*_*i*_) = *Q*_*i*_.
  If this is an *H*_2_ hash query on an identity *ID*_*i*_ and a message *M*_*i*_, then ℬ handles this query as follows: If the tuple (*ID*_*i*_, *M*_*i*_) already appears in the *H*_2_-list, then it responds with the value in the list. Otherwise, it randomly chooses $h_{i}\in {\mathbb{Z}}_{p}^{*}$, adds (*ID*_*i*_, *M*_*i*_, *h*_*i*_) to the *H*_2_-list, and responds with *H*_2_(*ID*_*i*_‖*M*_*i*_) = *h*_*i*_.
- **Private-Key Query**: ℬ handles a private key query for an identity *ID*_*i*_ as follows: It first retrieves (*ID*_*i*_, *t*_*i*_, *c*, *Q*_*i*_) from the *H*_1_-list. If *c* = 0, then it aborts the simulation since it cannot create a private key. Otherwise, it creates the private key $SK_{ID_{i}}=(D_{1}=g_{1}^{t_{i}},D_{2}=g_{2}^{t_{i}})$ and responds to 𝓐 with *SK*_*ID*_*i*__.
- **Signature Query**: ℬ handles a signature query on an identity *ID*_*i*_ and a message *M*_*i*_ as follows: It randomly chooses $r^{\prime }\in {\mathbb{Z}}_{p}^{*}$ and computes *h* = *H*_2_(*ID*_*i*_‖*M*_*i*_). Next, it responds to 𝓐 with a signature $\sigma =(U=g^{r^{\prime }}\cdot H_{1}{\left(ID_{i}\right)}^{-h},\;V=g_{1}^{r^{\prime }},\;W=H_{1}{\left(ID_{i}\right)}^{s_{2}}\cdot U^{s_{2}})$.
- **Output**: 𝓐 finally outputs a forged signature σ* = (U*, V*, W*) on an identity ID* and a message M*.

To solve the CDH problem, ℬ retrieves the tuple (ID*, t*, c*, Q*) from the *H*_1_-list. If c* ≠ 0, then it aborts since it cannot extract the CDH value. Otherwise, it obtains two valid signatures $\sigma_{1}^{*}=(U_{1}^{*},V_{1}^{*},W_{1}^{*})$ and $\sigma_{2}^{*}=(U_{2}^{*},V_{2}^{*},W_{2}^{*})$ on the same identity and message tuple (ID*, M*) such that $U_{1}^{*}=U_{2}^{*}$ and $h_{1}^{*}\neq h_{2}^{*}$ by applying Forking Lemma. That is, it replays ℱ with the same random tape but with a different choice of the random oracle *H*_2_. If $U_{1}^{*}=U_{2}^{*}$, then we have the following equation

$$\begin{array}{ccc} V_{1}^{*}\cdot {\left(V_{2}^{*}\right)}^{-1} & = & H_{1}{\left(ID^{*}\right)}^{s_{1}h_{1}^{*}}g_{1}^{s_{1}r^{*}}\cdot {\left(H_{1}{\left(ID^{*}\right)}^{s_{1}h_{2}^{*}}g_{1}^{s_{1}r^{*}}\right)}^{-1}\\ & = & H_{1}{\left(ID^{*}\right)}^{s_{1}\left(h_{1}^{*}-h_{2}^{*}\right)}={\left(g^{ab}\right)}^{t^{*}\left(h_{1}^{*}-h_{2}^{*}\right)}. \end{array}$$

Thus, ℬ can compute the CDH value as ${\left(V_{1}^{*}\cdot {\left(V_{2}^{*}\right)}^{-1}\right)}^{1/(t^{*}(h_{1}^{*}-h_{2}^{*}))}$ if $h_{1}^{*}\neq h_{2}^{*}\;mod\;p$.

### 4.2 A Non-Extractable Forgery

To extract the CDH value from forged signatures by applying Forking Lemma, it is essential for the simulator to obtain two valid signatures $\sigma_{1}^{*}$ and $\sigma_{2}^{*}$ such that $U_{1}^{*}=U_{2}^{*}$ and $h_{1}^{*}\neq h_{2}^{*}$. By replaying the forgery with the same random tape, but with a different choice of random oracle *H*_2_, it is possible for the simulator to obtain two valid signatures $\sigma_{1}^{*}=(U_{1}^{*},V_{1}^{*},W_{1}^{*})$ and $\sigma_{2}^{*}=(U_{2}^{*},V_{2}^{*},W_{2}^{*})$ with $h_{1}^{*}\neq h_{2}^{*}$ because of Forking Lemma. However, we show that the probability of $U_{1}^{*}=U_{2}^{*}$ can be negligible if the forgery is clever.

**Lemma 4.1** *If there is a PPT algorithm 𝓐 that can forge the IBS scheme of Yuan et al., then there is another PPT algorithm ℱ that can forge the IBS scheme with almost identical probability, but the simulator of Yuan et al. cannot extract the CDH value from the forged signatures of ℱ.*

*Proof*. The basic idea of this proof is that anyone can re-randomize the signature of Yuan et al.’s IBS scheme by using the public parameters. In this case, even though a simulator uses the same random tape for Forking Lemma, a forgery outputs a forged signature σ* on an identity ID* and a message M* after re-randomizing it by using the information h* = *H*_2_(ID*‖M*). Let *H*′:{0,1}* → ℤ_*p*_ be a collision resistant hash function that is not modeled as the random oracle. A new forgery ℱ that uses 𝓐 as a sub-routine is described as follows:

1. ℱ is first given *PP* and runs 𝓐 by giving *PP*. ℱ also handles the private key and signature queries of 𝓐 by using his own private key and signature oracles.
2. 𝓐 outputs a forged signature *σ*′ = (*U*′, *V*′, *W*′) on an identity ID* and a message M*.
3. ℱ computes h* = *H*_2_(ID*‖M*) and *h*′ = *H*′(*U*′‖h*), and then re-randomizes the forged signature of 𝓐 as
  $$\begin{array}{c} \sigma^{*}=(U^{*}=U^{\prime }\cdot g^{h^{\prime }},\;V^{*}=V^{\prime }\cdot g_{1}^{h^{\prime }},\;W^{*}=W^{\prime }\cdot g_{2}^{h^{\prime }}). \end{array}$$
4. Finally, ℱ outputs a forged signature σ* = (U*, V*, W*) on an identity ID* and M*.

To finish the proof, we should show that the forged signature of ℱ is correct and the simulator of Yuan et al. cannot extract the CDH value from the forged signatures by using Forking Lemma. Let *r*′ be the randomness of *σ*′. The correctness of the forged signature is easily checked as follows

$$\begin{array}{ccc} U^{*} & = & U^{\prime }\cdot g^{h^{\prime }}=g^{r^{\prime }+h^{\prime }}=g^{r^{*}},\\ V^{*} & = & V^{\prime }\cdot g_{1}^{h^{\prime }}=H_{1}{\left(ID^{*}\right)}^{s_{1}h^{*}}g_{1}^{r^{\prime }+h^{\prime }}=H_{1}{\left(ID^{*}\right)}^{s_{1}h^{*}}g_{1}^{r^{*}},\\ W^{*} & = & W^{\prime }\cdot g_{2}^{h^{\prime }}=H_{1}{\left(ID^{*}\right)}^{s_{2}}g_{2}^{r^{\prime }+h^{\prime }}=H_{1}{\left(ID^{*}\right)}^{s_{2}}g_{2}^{r^{*}} \end{array}$$

where h* = *H*_2_(ID*‖M*) and r* = *r*′+*h*′. To extract the CDH value from the forged signature of ℱ by using Forking Lemma, the simulator of Yuan et al. should obtain two valid signatures $\sigma_{1}^{*}=(U_{1}^{*},V_{1}^{*},W_{1}^{*})$ and $\sigma_{2}^{*}=(U_{2}^{*},V_{2}^{*},W_{2}^{*})$ on the same identity and message pair (ID*, M*) such that $U_{1}^{*}=U_{2}^{*}$ and $h_{1}^{*}\neq h_{2}^{*}$ after replaying ℱ with the same random tape but with a different choice of the hash oracle *H*_2_. Let $\sigma_{1}^{*}=(U_{1}^{*},V_{1}^{*},W_{1}^{*})$ and $\sigma_{2}^{*}=(U_{2}^{*},V_{2}^{*},W_{2}^{*})$ be the two valid signatures obtained from ℱ by using Forking Lemma. Let $\sigma_{{}^{{}_{1}}}^{\prime }=(U_{{}^{{}_{1}}}^{\prime },V_{{}^{{}_{1}}}^{\prime },W_{{}_{1}}^{{}^{\prime }})$ and $\sigma_{{}^{{}_{2}}}^{\prime }=(U_{{}^{{}_{2}}}^{\prime },V_{{}^{{}_{2}}}^{\prime },W_{{}^{{}_{2}}}^{\prime })$ be the original signatures before the re-randomization of ℱ. If $h_{1}^{*}\neq h_{2}^{*}$, then we have $h_{{}^{{}_{1}}}^{\prime }\neq h_{{}^{{}_{1}}}^{\prime }$ with non-negligible probability since *H*′ is a collision-resistance hash function and the inputs of the hash function are different. From $h_{{}^{{}_{1}}}^{\prime }\neq h_{{}^{2}}^{\prime }$, we have $U_{1}^{*}\neq U_{2}^{*}$ with non-negligible probability since $U_{{}^{{}_{1}}}^{\prime }$ and $U_{{}^{{}_{2\;}}}^{\prime }$ are re-randomized with difference values $g^{h_{{}^{{}_{1}}}^{\prime }}$ and $g^{h_{2}^{\prime }}$ respectively. Therefore, the event of obtaining two valid signatures only occurs with negligible probability. This completes our proof.

### 4.3 Discussions

From the above analysis, we know that the security of the original IBS scheme of Yuan et al. cannot be proven under the CDH assumption by applying Forking Lemma since the forger can easily re-randomize the signatures. To fix this problem, we may modify the IBS scheme to compute *h* = *H*_2_(*U*‖*ID*‖*M*) instead of computing *h* = *H*_2_(*ID*‖*M*) where *U* is the first element of a signature. In this case, the forger cannot re-randomize the signature of the modified IBS scheme since *U* is an input of *H*_2_. Unfortunately, this modified IBS scheme cannot be used to construct an IBAS scheme since the *U* from the individual signatures cannot be aggregated. Note that if each *U* is aggregated, then a verifier cannot check the validity of an aggregate signature since each *U* is not included in the aggregate signature. Therefore, there is no easy solution to the problem.

## 5 Conclusion

In this paper, we showed that the IBAS scheme of Yuan et al. that achieves both full aggregation and constant pairing computation is not secure at all. We presented an efficient forgery algorithm against the IBS scheme and showed that their security proof of the scheme has a serious flaw. To invalidate the security of their IBS scheme, we showed that a linear combination of two valid signatures is also a new signature. Since the security of their IBAS scheme is based on the security of their IBS scheme, their IBAS scheme is also insecure. Therefore, constructing an IBAS scheme with full aggregation in bilinear maps still remains an important open problem.
